# Effect of gene, environment and maternal depressive symptoms on pre-adolescence behavior problems – a longitudinal study

**DOI:** 10.1186/1753-2000-7-10

**Published:** 2013-03-22

**Authors:** Sara Agnafors, Erika Comasco, Marie Bladh, Gunilla Sydsjö, Linda DeKeyser, Lars Oreland, Carl Göran Svedin

**Affiliations:** 1 Division of Child and Adolescent Psychiatry, IKE, Faculty of Health Sciences, Linköping University, Linköping, S-581 85, Sweden; 2 Division of Pharmacology, Department of Neuroscience, Uppsala University, BMC, Box 593,, Uppsala, S-751 24, Sweden; 3 Division of Obstetrics and Gynecology IKE, Faculty of Health Sciences, Linköping University, Linköping, S-581 85, Sweden

**Keywords:** Child, Depression, *5-*HTTLPR, *BDNF*, Longitudinal, SESBiC-study

## Abstract

**Background:**

Depression is a common and disabling condition with a high relapse frequency. Maternal mental health problems and experience of traumatic life events are known to increase the risk of behavior problems in children. Recently, genetic factors, in particular gene-by-environment interaction models, have been implicated to explain depressive etiology. However, results are inconclusive.

**Methods:**

Study participants were members of the SESBiC-study. A total of 889 mothers and their children were followed during the child’s age of 3 months to 12 years. Information on maternal depressive symptoms was gathered postpartum and at a 12 year follow-up. Mothers reported on child behavior and traumatic life events experienced by the child at age 12. Saliva samples were obtained from children for analysis of *5-*HTTLPR and *BDNF* Val66Met polymorphisms.

**Results:**

Multivariate analysis showed a significant association between maternal symptoms of depression and anxiety, and internalizing problems in 12-year-old children (OR 5.72, 95% CI 3.30-9.91). Furthermore, carriers of two short alleles (s/s) of the *5*-HTTLPR showed a more than 4-fold increased risk of internalizing problems at age 12 compared to l/l carriers (OR 4.73, 95% CI 2.14-10.48). No gene-by-environment interaction was found and neither depressive symptoms postpartum or traumatic experiences during childhood stayed significant in the final model.

**Conclusions:**

Concurrent maternal symptoms of depression and anxiety are significant risk factors for behavior problems in children, which need to be taken into account in clinical practice. Furthermore, we found a main effect of *5-*HTTLPR on internalizing symptoms in 12-year-old children, a finding that needs to be confirmed in future studies.

## Background

Depression and anxiety conditions are an increasing problem, leading to substantial economic and social consequences [1]. The prevalence of depression in pre-adolescents is approximately 1% [2] and there is an increase in rates of various psychiatric problems during adolescence and adulthood in individuals with a history of childhood depression [3,4]. Moreover, childhood depression has shown a risk factor pattern different from that of adolescent and adult onset depression, raising the question whether childhood depression is etiologically separate from the latter [5,6].

The longitudinal approach serves as an excellent way of studying risk factors, development and course of psychiatric disorders and symptoms. Many factors may elevate the risk for onset of depression and emotional problems in children. Postpartum depression has been shown to predict child behavior problems in numerous studies [7,8], but there is also support for the fact that on-going maternal depression exerts a risk factor for internalizing as well as externalizing symptoms in children [9-11].

Among other known risk factors for depression and emotional problems are traumatic life events [12] - children exposed to physical abuse, parental divorce and domestic violence are at increased risk of behavior problems and adult depression [13-15]. Still, not everyone subjected to traumatic life events develops depressive symptoms. Hence, there may be individuals predisposed or more vulnerable to certain risk factors.

In recent years, much effort has been given to explaining depression in relation to genetic factors. Many candidate genes have been examined, of which the serotonin transporter gene (*5-HTT)* is the most studied. The *5-HTT* gene includes a functional polymorphism, the serotonin transporter gene-linked polymorphic region (*5*-HTTLPR), which consists of two common alleles; short (s) and long (l). Less effective serotonin expression and availability have been shown in s-allele carriers, compared to individuals homozygous for the l allele [16]. The gene-by-environment model put up by Caspi *et al.*[17], showing an association between the *5*-HTTLPR, stressful life events and depression, has since publication been subjected to numerous attempts at replication. Some researchers have been able to reproduce the results partially or completely [18-24], whereas others have not [25-28], however between-study heterogeneity has to be taken into account. When looking particularly at *5*-HTTLPR gene-by-environment studies on children, research is not that extensive and results have been conflicting. Araya *et al.*[7] found no association between *5*-HTTLPR and emotional symptoms in seven-year-olds whereas associations have been shown by other researchers, although on smaller study samples [20,22,29,30]. Moreover, also direct association between *5*-HTTLPR and depression has been found [16,31-34].

Given not only the inconsistency of single genetic explanatory models, but also the complex etiology of depression, researchers have lately been looking at gene-by-gene (−by-environment) models for an explanation of depressive vulnerability. Another candidate gene for depressive disorders is the Brain Derived Neurotrophic Factor (*BDNF*), which has previously been shown to act in synergy with serotonin [35]. *BDNF* is involved in reparation, plasticity and neurogenesis in the brain, and a single nucleotide polymorphism (SNP) G/A (Val66Met) in the *BDNF* gene has been shown to affect levels of *BDNF* in the brain [36]. Similarly to *5*-HTTLPR, studies regarding the association between *BDNF* Val66Met and depression have been both confirmative [37,38] and negating [39].

A candidate gene-by-gene-by-environment interaction effect of *5*-HTTLPR-by-*BDNF*Val66Met-by-childhood adversity on depression has been shown [40] and to our knowledge four studies have attempted replication of this three-way interaction effect, however with contradictory results [41-44]. The findings are inconsistent regarding both the presence of a three-way interaction effect and the risk genetic variants in the presence of childhood adversity. Two studies found evidence of a three-way interaction effect [42,44] whereas two other studies did not [41,43].

In view of this, the aim of the present paper was to study the gene-by-environment interaction on depressive symptoms in 12-year-old children. We hypothesized to find a gene-by-environment, and possibly gene-by-gene-by-environment interaction on depressive symptoms, including the genetic polymorphisms *5*-HTTLPR and *BDNF* Val66Met, traumatic life events and maternal symptoms of depression.

## Methods

### Population and procedures

This study is part of the South East Sweden Birth Cohort study (SESBiC-study) which started in 1995 with the purpose of early identification of psychosocially burdened families where children were at risk of dysfunctional development. Follow-ups have been carried out at ages 3, 5.5 and 12 and have been reported previously [11,45-48].

#### Baseline

All mothers of children from a birth cohort born between May 1st 1995 and December 31st 1996 in southern Sweden were asked to take part in the study, of whom 1723 mothers (88%) agreed to participate. The mean age of the mothers was 28.2 ± 4.6 years at childbirth. Ninety six percent of the mothers (n=1574) were cohabitating, 3.5% (n=57) were single parents, while 0.5% (n=8) reported other family arrangements. Most mothers were born in Sweden (88.6%), (n=1482), but 6.2% (n=103) were born in Europe (excluding Sweden), and 5.3% (n=88) outside Europe. Of the newborn children, 52.8% were boys and there were 27 twin pairs. The baseline study was carried out at Child Welfare Centers, (CWC) in connection with the routine 3-month check-up. Questionnaires were administered and a psychologist also interviewed the mothers.

#### 12-year follow-up

Current home addresses for all 1 723 families were obtained from the Swedish Tax Offices. An information letter and a consent form were sent to parents (i.e. legal guardians). Parents who did not return the consent form within three weeks were contacted by phone. A separate, simplified information letter was enclosed for the child. Two children and four mothers were deceased, ten had moved out of the country and 24 were learning disabled and could therefore not participate. These subjects were excluded from the original 1723 in the baseline study, which left 1687 eligible participants, of whom 889 (52.7%) accepted participation. The follow-up was carried out at school where research assistants met with the children in small groups. The children provided saliva samples and filled out a package of questionnaires separately (as part of a larger study). A package of questionnaires was sent to the mothers’ home addresses. Families who had moved out of the original catchment area were contacted by mail and phone in accordance with the regular routine. Those who agreed to participate received questionnaires and saliva sampling kits by mail, or if they preferred, were visited by a research assistant and the survey was carried out at the child’s home.

### Genetic analyses

The non-invasive and all-in-one Oragene® DNA Collection Kit (DNA Genotek) was used for the collection, stabilization and transportation of saliva samples. DNA was isolated according to the laboratory protocol for manual purification of DNA.

*BDNF* Val66Met A/G SNP (rs6265) and *5*-HTTLPR genotyping were carried out according to previously published protocols [49]. The genotyping was performed blind to psychosocial data. To estimate the quality-rate of genotyping errors, a random repetition of ~13% of the sample was carried out; the comparison indicated no inconsistencies. The genotypes were in Hardy-Weinberg equilibrium (5-HTTLPR (*χ*^*2*^ =2.73; p=0.10); females (*χ*^*2*^=0.44; p=0.51); males (*χ*^*2*^=2.67; p=0.10), *BDNF* Val66Met (*χ*^*2*^=0.08; p=0.77); females (*χ*^*2*^=1.20; p=0.27); males (*χ*^*2*^=1.98; p=0.16)).

### Questionnaires

#### Baseline

The Edinburgh Postnatal Depression Scale (EPDS) [50] is a widely used self-report questionnaire designed to screen for post-natal depression. EPDS refers to the 7 days preceding completion of the form and was filled out by the mothers at baseline.

Life Stress Score (LSS) is a 50-item semi-structured interview form, consisting of three main domains regarding the mother’s social situation, medical information and psychological circumstances. The LSS has been used previously in a Swedish population based study [51] and was filled out by a psychologist after interviewing the mothers at baseline.

#### 12-year follow-up

The Child Behavior Check List (CBCL) [52] is a 113-item form assessing child behavior, focusing on subscales of internalizing and externalizing behavior respectively. We used the CBCL 4–18 years, which was filled out by the mothers at the child’s age of 12.

The Hopkins Symptom Checklist (HSCL-25), a short version of the HSCL-90 [53] was used to measure symptoms of anxiety and depression during the most recent 14 days. The HSCL-25 was filled out by the mothers at the 12-year follow-up.

To assess potentially traumatic life events experienced by the child, the Swedish version of Life Incidence of Traumatic Events (LITE) was used [54,55]. The 16-item parent report form (LITE-P) was filled out by the mothers at the child’s age of 12.

### Data analysis

In accordance with previous studies, we used two cut offs for the EPDS in order to catch depressive symptoms of different severity. The first (cut off 10) representing a screening level [50] and the second (cut off 13) supposed to catch more severe clinical depressive symptomatology [56]. For the HSCL-25 total score, a mean item score of 1.75 was used as cut off, as has been used previously [57]. On the CBCL and LSS scales, the 90th percentile was set as a cut off. Results on the LITE form were dichotomized into 0–2 events and ≥3 events, for which there is support in the literature [17]. Bivariate analyses between genetic markers (*5*-HTTLPR and *BDNF* Val66Met) and psychological scales (CBCL), as well as between scales (EPDS, HSCL-25, CBCL), were performed using the chi-square statistic. Multivariate analyses, with CBCL scales as dependent variables and psychological scales, socio-demographic variables (LSS) and genetic markers as independent variables were also performed. Ethnic background (both parents born in Sweden, compared to one or both parents born abroad) and sex of the child were also controlled for. The multivariate analysis consisted of conditional stepwise logistic regression considering full factorial models. However, since this procedure may choose models containing interactions without corresponding main effects, the models have been corrected for this and further evaluated and reduced to include models with significant main effects and appropriate corresponding interactions. Results are presented with corresponding Odds Ratios (OR) and 95% Confidence Intervals (CI). All statistical analyses were performed using IBM SPSS version 19 (IBM Corporation, Armonk, NY).

### Dropout rate analysis

At the 12-year follow-up, the total dropout rate was 47.3% (n=798). There was a difference when comparing immigrant status between participants and non-participants at the 12 year follow-up, where 54.6% (n=802) of mothers born in Sweden (n=1468) took part compared to 44.6% (n=45) of mothers born in Europe (n=101) and 34.9% (n=30) of mothers born outside of Europe (n=86) (χ2=15.79, p=0.00). Likewise, differences were found between participants and non-participants at the follow-up when comparison for socio-demographic factors at baseline was made. Of mothers who scored above cut-off on the LSS total scale at baseline (n=141), 60.3% (n=85) took part in the follow-up compared to 70.7% (n=1093) of mothers with low total score at baseline (n=1546) (χ2=6.65, p=0.01). No differences were found between participants and non-participants at the 12-year follow-up regarding symptoms of postpartum depression.

### Ethical approval

The study outline was approved by the Ethics committee at the University of Lund in 1994 and 1998 and by The Regional Ethical Review Board in Linköping 2007.

## Results

The frequencies of *5*-HTTLPR genotypes were l/l 31%, s/l 47% and s/s 22%. The frequencies of *BDNF* Val66Met genotypes were Val/Val 65%, Val/Met 31%, Met/Met 4%.

### Bivariate analysis

In bivariate analysis, maternal symptoms of depression and anxiety at the 12-year follow up increased the risk for internalizing problems in the children more than fivefold (OR 5.92, CI 3.59-9.77). There was an increase of risk also for the subscales anxious depressed and withdrawn depressed (OR 4.76, CI 2.95-7.70; OR 4.54, CI 2.82-7.30). A fivefold risk increase was seen for externalizing problems (OR 5.20, CI 3.26-8.28) (Table 1).

**Table 1:** Odds ratios in predicting CBCL subscales at age 12

	**Odds ratio**	**95.0% CI for odds ratio**	**p-value**
**Internalizing symptoms ≥ 90th percentile**			
BDNF Val/Met compared to Val/Val	1.37	0.82-2.29	0.24
BDNF Met/Met compared to Val/Val	0.82	0.19-3.52	0.78
5HTTLPR s/l compared to l/l	1.78	0.84-3.76	0.13
5HTTLPR s/s compared to l/l	4.43	2.09-9.38	<0.01
LITE	1.70	1.04-2.80	0.04
EPDS screening cut off 10	1.90	0.98-3.68	0.06
Clinical cut off 13	3.09	1.30-7.34	0.01
HSCL-25	5.92	3.59-9.77	<0.001
LSS	4.03	1.95-8.35	<0.001
Sex	0.63	0.38-1.04	0.07
Ethnicity	1.93	1.02-3.67	0.04
**Anxious depressed ≥ 90th percentile**			
BDNF Val/Met compared to Val/Val	0.99	0.59-1.64	0.96
BDNF Met/Met compared to Val/Val	0.64	0.15-2.76	0.55
5HTTLPR s/l compared to l/l	1.10	0.59-2.06	0.76
5HTTLPR s/s compared to l/l	2.32	1.22-4.42	0.01
LITE	1.72	1.07-2.72	0.03
EPDS screening cut off 10	1.84	0.97-3.49	0.06
Clinical cut off 13	2.72	1.15-6.43	0.02
HSCL-25	4.76	2.95-7.70	<0.001
LSS	3.53	1.71-7.28	0.001
Sex	0.59	0.37-0.96	0.03
Ethnicity	1.19	0.59-2-39	0.63
**Withdrawn depressed ≥ 90th percentile**			
BDNF Val/Met compared to Val/Val	1.53	0.95-2.51	0.80
BDNF Met/Met compared to Val/Val	0.75	0.17-3.26	0.70
5HTTLPR s/l compared to l/l	1.27	0.68-2.38	0.46
5HTTLPR s/s compared to l/l	2.48	1.29-4.77	0.07
LITE	1.32	0.83-2.10	0.24
EPDS screening cut off 10	2.04	1.09-3.81	0.03
Clinical cut off 13	2.68	1.13-6.34	0.02
HSCL-25	4.54	2.82-7.30	<0.001
LSS	1.52	0.62-3.69	0.36
Sex	1.28	0.81-2.03	0.30
Ethnicity	2.22	1.22-4.01	0.01
**Externalizing symptoms ≥ 90th percentile**			
BDNF Val/Met compared to Val/Val	1.28	0.78-2.10	0.33
BDNF Met/Met compared to Val/Val	1.50	0.51-4.46	0.47
5-HTTLPR s/l compared to l/l	1.60	0.86-3.01	0.14
5-HTTLPR s/s compared to l/l	2.40	1.23-4.75	0.01
LITE	1.62	1.02-2.56	0.04
EPDS screening cut off 10	2.07	1.13-3.80	0.02
Clinical cut off 13	2.52	1.07-5.97	0.03
HSCL-25	5.20	3.26-8.28	<0.001
LSS	2.07	0.93-4.62	0.08
Sex	1.86	1.17-2.95	0.01
Ethnicity	1.89	1.04-3.46	0.04

Note: CI = Confidence Interval, CBCL = Child Behavior Checklist, BDNF = Brain Derived Neurotrophic Factor, 5-HTTLPR = serotonin transporter gene-linked polymorphic region, LITE = Life Incidence of Traumatic Events, EPDS = Edinburgh Postnatal Depression Scale, HSCL-25 = Hopkins Symptom Checklist 25, LSS = Life Stress Score.

Logistic regression. Dependent variables: CBCL (internalizing symptoms, internalizing symptoms divided into 1) anxious depressed and 2) withdrawn depressed, and externalizing symptoms). Independent variables (0 is used as a reference level): LITE (0≤ 2, 1≥3), EPDS (0≤9, 1≥10) and (0≤12, 1≥13), HSCL-25 (0=mean score <1.75, 1= mean score ≥1.75): LSS (0<90th percentile, 1≥90th percentile) sex (0=girls and 1=boys) and ethnicity (0=Swedish 1=2nd generation immigrants).

Using the screening cut off of 10 on EPDS no effect was seen for maternal depressive symptoms postpartum on internalizing problems and the subscale anxious depressed in the children. There was an increase of risk for withdrawn depressive symptoms (OR 2.04, CI 1.09-3.81) and externalizing symptoms (OR 2.07, CI 1.13-3.80) if the mother reported on depressive symptoms postpartum (Table 1). Using the higher cut off of 13 reduced the number of EPDS positive mothers from 92 to 35. The association between maternal depressive symptoms postpartum and all CBCL scales were strengthened using the clinical cut off of 13 on the EPDS (Table 1).

Analysis further showed that *5*-HTTLPR s/s carriers had more internalizing problems at age 12 compared to l/l carriers (OR 4.43, CI 2.09-9.38). The same applied for the subscales anxious depressed and withdrawn depressed (OR 2.32, CI 1.22-4.42; OR 2.48, CI 1.29-4.77) as well as for externalizing problems (OR 2.4, CI 1.23-4.75). Differences between s/l and l/l carriers were not significant. No effect was seen for *BDNF* Val66Met genotype on child behavior problems (Table 1).

Traumatic life events increased the risk for internalizing and externalizing problems in the children (OR 1.70, CI 1.04-2.80; OR 1.62, CI 1.02-2.56). For the subscale anxious depressed, there was an increase of risk (OR 1.72, CI 1.07-2.72), but not concerning the subscale withdrawn depressed (Table 1).

Maternal life stress at baseline increased the risk for internalizing problems in the children (OR 4.03, CI 1.95-8.35). A risk increase was also seen for the subscale anxious depressed (OR 3.53, CI 1.71-7.28), but not for withdrawn depressed and externalizing problems (Table 1).

Children with a non-Swedish background had an increased risk for both internalizing and externalizing problems (OR 1.93, CI 1.02-3.67; OR 1.89, CI 1.04-3.46) compared to children with a Swedish background. They also indicated an increased risk on the subscale withdrawn depressed (OR 2.22, CI 1.22-4.01) (Table 1). Boys had an increased risk for externalizing problems compared to girls (OR 1.86, CI 1.17-2.95), whereas girls had an increased risk on the subscale anxious depressed (OR 0.59, CI 0.37-0.96) (Table 1).

### Multivariate analysis

**Figure 1:**
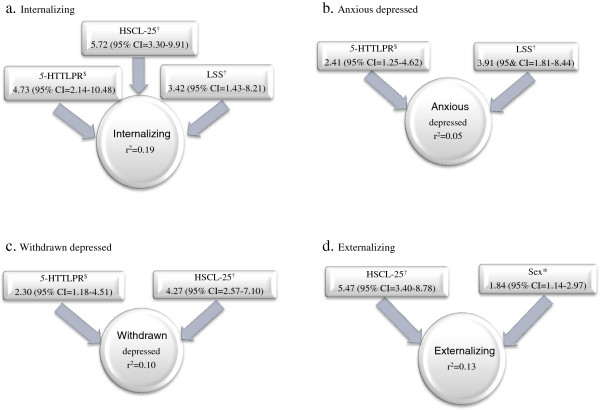
**Odds ratios for the studied CBCL outcomes and corresponding measure of r2.** $Comparing s/s with l/l as reference. †Comparing risk with no risk as reference. *Comparing boys with girls as reference. Note: CBCL = Child Behavior Checklist, r^2 =^ the amount of variance explained by the model, 5-HTTLPR = serotonin transporter gene-linked polymorphic region, HSCL-25 = Hopkins Symptom Checklist 25, LSS = Life Stress Score. Multivariate analysis. CBCL scales presented are **a**) internalizing symptoms, internalizing subscales **b**) anxious depressed and **c**) withdrawn depressed, and **d**) externalizing symptoms.

In the stepwise multivariate analysis using cut off of 10 on EPDS, maternal symptoms of depression and anxiety at the child’s age of 12 increased the risk for both internalizing and externalizing behavior problems in the children (OR 5.72, CI 3.30-9.91; OR 5.47, CI 3.40-8.78). When dividing the internalizing scale into the subscales anxious depressed and withdrawn depressed, the effect remained only for the latter (OR 4.27, CI 2.57-7.10) (Figure 1).

*5*-HTTLPR s/s carriers had a more than 4-fold increased risk of internalizing problems at age 12 compared to l/l carriers (OR 4.73, CI 2.14-10.48). Likewise, a risk increase was seen for the subscales anxious depressed and withdrawn depressed (OR 2.41, CI 1.25-4.62; OR 2.30, CI 1.18-4.51) (Figure 1). Differences between s/l and l/l carriers were not significant. The *BDNF* Val66Met polymorphism did not show an impact on child behavior, and was not included in the final models.

Maternal life stress at baseline increased the risk for internalizing problems in 12-year old children (OR 3.42, CI 1.43-8.21). This was true also for the subscale anxious depressed (OR 3.91, CI 1.81-8.44) (Figure 1). No effect was seen for LITE on any of the models.

Boys had an increased risk of externalizing problems compared to girls (OR 1.84, CI 1.14-2.97). No other differences relating to sex were detected.

The only change when using a cut off of 13 on EPDS was seen concerning the outcome in the model for the subscale anxious depressed. The significance of 5-HTTLPR disappeared while both being girl (OR 0.53, CI 0.32-0.87) and maternal symptoms of depression and anxiety at the child’s age of 12 (OR 4.62 CI 2.81-7.58) increased the risk for being anxious depressed according to the CBCL subscale. No separate significant effect was seen for EPDS using either of the two cut offs.

There was no evidence for gene-by-environment interactions, or gene-by-gene-by-environment interactions. Furthermore, we tested whether there was an interaction between ethnic background and *5-*HTTLPR and *BDNF* Val66Met polymorphisms respectively on the outcome of child behavior problems, but no such interaction was found (for all factors and interactions tested, see Additional file 1).

## Discussion

This study used data from a longitudinal birth cohort study in order to examine predictors of internalizing and externalizing symptoms in 12-year old children. Environmental as well as genetic factors were included in order to test for gene-by-environment and gene-by-gene-by-environment interactions. The results of the study can be summarized in three main findings.

First and foremost, maternal symptoms of depression and anxiety predicted child behavior problems at age 12. Numerous researchers have been able to show the importance of maternal mental health for child development and wellbeing. Our results indicate that in the present population, maternal symptoms of postpartum depression (analyzed with different cut off scores on the EPDS) did not have a long-term impact on child behavior, i.e., no increase of risk was seen at age 12. Symptoms of depression in mothers at the 12-year follow-up could possibly reflect recurrent or chronic depressive problems, which would put the child under greater stress than would be the case with a single episode depression. In clinical practice, we suggest that besides focusing on the child’s mental health, it is important also to ask about depressive symptoms in the mother.

Second, we found a main gene effect of *5-*HTTLPR on child behavior at age 12. Since the finding of an interaction between *5*-HTTLPR and stressful life events [17], focus of research has shifted to gene-by-environment interaction effects. The present finding does have support in the literature [16,31-34], however most gene-by-environment studies have not shown a main effect of *5*-HTTLPR on depression [20,21]. Recently, Duncan and Keller reviewed the literature on candidate gene-by-environment interaction (cGxE) studies in psychiatry over a decade (2000–2009), and based on the analyses of 103 studies suggested that “well-powered direct replications deserve more attention than novel cG×E findings and indirect replications” [58]. The current study attempted testing a previous hypothesis and replicating previous findings in a large and longitudinal population-based independent study/sample. The set-up of the current study can help to understand the generalizability of previous findings since it used a virtually similar set-up compared to previous studies with regard to phenotypic variables, genetic polymorphisms, statistical model, environmental moderator, and inclusion of both sexes [58]. This represents a major strength of the current study which might contribute to the drawing of clearer conclusions in the context of future meta-analytical studies. The present study did not find any GxE or GxGxE effects but rather a main effect of *5-*HTTLPR on internalizing symptoms as for example showed by Hoefgren et al. in a case–control study on depression [31].

Third, traumatic life events showed an impact on child behavior in bivariate models, but not in the multivariate analyses, indicating that in this material, life events did not explain as much of the variance as other factors. We used the mothers’ reports on traumatic life events experienced by the children. Previous studies have shown differences between parents’ reports and children’s self-reports on exposure to violence [59]. Considering this, self-reports might have contributed to a more fair estimation of traumatic life events experienced by the child. Given the known relationship between life events and depressive symptoms, one would have expected an effect on the internalizing scales [12,15,17].

We included measure of externalizing problems since previous research has shown high comorbidity between depressive and externalizing disorders in adolescents [60] and since boys more often express their psychological problems or stress by showing externalizing problems [2]. Furthermore, Laucht *et al.*[61] reported that adolescents with depressive disorders were more likely to have a history of externalizing problems. As expected, the analysis showed that boys expressed significantly more externalizing problems than girls, but there were no differences relating to sex for internalizing problems.

Despite the dropout level, the number of its participants and the longitudinal approach strengthens the study. There are some limitations that need to be mentioned. Although dropout rates of the same magnitude in longitudinal, multi wave studies are common [7], the skewed dropout in this study is a limitation - psychosocially disadvantaged families and families of foreign origin were less likely to participate in the follow-up. Considering the known connection between psychosocial strain and behavior problems, it is plausible to think that a more representative participating population would have strengthened the results.

In general, both child and parent-reports indicated low frequencies of behavior problems. Reports on good mental health in 12-year old children have been published previously. Costello *et al.*[2] examined mental health in 9–16 year old children using parent’s reports on child behavior (CBCL) and clinical interviews, and found the lowest prevalence of depression in 12-year olds (0.4%). Conducting the follow-up at ages eight-ten or during adolescence, where previous reports have shown more behavior problems, might have increased the prevalence rate and thereby rendering a larger group for different analyses.

Since there are studies indicating that depressed mothers are prone to overestimate behavior problems in their children [62], another limitation of the present study is that mothers’ reports, not self-reports or teachers' reports, on child behavior and traumatic life events were used. We decided to use mothers as informants since they have known their children for 12 years around the clock compared to the current teachers that have known the children for about just over a year 5–6 hours a day, five days a week. Another support for this strategy was that although the association between maternal depressive symptoms and a positive mother–child reporting discrepancy has been demonstrated, in the general population this association is small and does not bias research, using maternal reports on child problems [62]. The chosen methodology used in the study also allowed following data from the same informants from baseline to the 12-year follow-up.

Feder, Nestler and Charney’s report of 2009 called for an increased understanding of the psycho-biological factors which are involved in risk and resiliency for psychiatric disorders [63], and Duncan and Keller‘s review called for well-powered direct replications of cG×E findings [58]. Thus, many studies are still to be carried out to shed light on the psychogenetic underpinning of depression.

## Conclusion

In conclusion the present study suggests that concurrent maternal symptoms of depression and anxiety are an important risk for behavior problems in children, which needs to be taken into account in clinical practice. Furthermore, we found a main effect of *5-*HTTLPR on internalizing symptoms in 12 year old children, a finding that need to be confirmed in future studies.

## Competing interests

The authors declare that they have no competing interests.

## Authors’ contributions

SA was responsible for the data collection, and for writing the manuscript. EC was responsible for the genetic analyses, and made substantial contribution to the writing of the manuscript. MB was responsible for the statistical analyses, and took part in writing the manuscript. LD took part in data collection and writing the manuscript. CGS and GS planned and supervised the research project. LO took part in planning the project and supervised the genetic analyses. All authors took part in reviewing draft versions of the manuscript, and approved of the final version.

## Supplementary Material

Additional file 1 **Factors and interactions included in multivariate analysis.** The additional file holds a list of all factors and interactions between factors tested in multivariate analysis. Click here for file

## References

[B1] Johnston K Westerfield W Momin S Philippi R Naldoo A The direct and indirect costs of employee depression, anxiety, and emotional disorders - an employer case study J Occup Environ Med 2009 51 564 577 10.1097/JOM.0b013e3181a1f5c8 19369892

[B2] Costello EJ Mustillo S Erkanli A Keeler G Angold A Prevalence and development of psychiatric disorders in childhood and adolescence Arch Gen Psychiatry 2003 60 8 837 844 10.1001/archpsyc.60.8.837 12912767

[B3] Kim-Cohen J Caspi A Moffitt TE Harrington H Milne BJ Poulton R Prior juvenile diagnoses in adults with mental disorder: developmental follow-back of a prospective-longitudinal cohort Arch Gen Psychiatry 2003 60 7 709 717 10.1001/archpsyc.60.7.709 12860775

[B4] Copeland WE Shanahan L Costello EJ Angold A Childhood and adolescent psychiatric disorders as predictors of young adult disorders Arch Gen Psychiatry 2009 66 7 764 772 10.1001/archgenpsychiatry.2009.85 19581568 PMC2891142

[B5] Jaffee SR Moffitt TE Caspi A Fombonne E Poulton R Martin J Differences in early childhood risk factors for juvenile-onset and adult-onset depression Arch Gen Psychiatry 2002 59 3 215 222 10.1001/archpsyc.59.3.215 11879158

[B6] Hill J Pickles A Rollinson L Davies R Byatt M Juvenile- versus adult-onset depression: multiple differences imply different pathways Psychol Med 2004 34 8 1483 1493 10.1017/S0033291704002843 15724879

[B7] Araya R Hu X Heron J Enoch MA Evans J Lewis G Nutt D Goldman D Effects of stressful life events, maternal depression and 5-HTTLPR genotype on emotional symptoms in pre-adolescent children Am J Med Genet B Neuropsychiatr Genet 2009 150B 5 670 682 10.1002/ajmg.b.30888 19016475 PMC4392724

[B8] Fihrer I McMahon CA Taylor AJ The impact of postnatal and concurrent maternal depression on child behavior during the early school years J Affect Disord 2009 119 116 123 10.1016/j.jad.2009.03.001 19342104

[B9] Hammen C Brennan PA Shih JH Family discord and stress predictors of depression and other disorders in adolescent children of depressed and nondepressed women J Am Acad Child Adolesc Psychiatry 2004 43 8 994 1002 10.1097/01.chi.0000127588.57468.f6 15266194

[B10] Luoma I Tamminen T Kaukonen P Laippala P Puura K Salmelin R Almqvist F Longitudinal study of maternal depressive symptoms and child well-being J Am Acad Child Adolesc Psychiatry 2001 40 12 1367 1374 10.1097/00004583-200112000-00006 11765281

[B11] Agnafors S Sydsjö G DeKeyser L Svedin CG Symptoms of Depression Postpartum and 12 years Later-Associations to Child Mental Health at 12 years of Age Matern Child Health J 2012 [Epub ahead of print] 10.1007/s10995-012-0985-z 22466717

[B12] Tennant C Life events, stress and depression: a review of recent findings Aust N Z J Psychiatry 2002 36 2 173 182 10.1046/j.1440-1614.2002.01007.x 11982537

[B13] Rønning JA Haavisto A Nikolakaros G Helenius H Tamminen T Moilanen I Kumpulainen K Piha J Almqvist F Sourander A Factors associated with reported childhood depressive symptoms at age 8 and later self-reported depressive symptoms among boys at age 18 Soc Psychiatry Psychiatr Epidemiol 2011 46 3 207 218 10.1007/s00127-010-0182-6 20145907

[B14] Chapman DP Whitfield CL Felitti VJ Dube SR Edwards VJ Anda RF Adverse childhood experiences and the risk of depressive disorders in adulthood J Affect Disord 2004 82 2 217 225 10.1016/j.jad.2003.12.013 15488250

[B15] Carter AS Wagmiller RJ Gray SA McCarthy KJ Horwitz SM Briggs-Gowan MJ Prevalence of DSM-IV disorder in a representative, healthy birth cohort at school entry: sociodemographic risks and social adaptation J Am Acad Child Adolesc Psychiatry 2010 49 7 686 698 20610138 10.1016/j.jaac.2010.03.018 PMC3166638

[B16] Lesch KP Bengel D Heils A Sabol SZ Greenberg BD Petri S Benjamin J Muller CR Hamer DH Murphy DL Association of anxiety-related traits with a polymorphism in the serotonin transporter gene regulatory region Science 1996 274 1527 1531 10.1126/science.274.5292.1527 8929413

[B17] Caspi A Sugden K Moffit TE Taylor A Craig IW Harrington H Influence of life stress on depression: Moderation by a polymorphism in the 5-HTT gene Science 2003 301 386 389 10.1126/science.1083968 12869766

[B18] Wilhelm K Mitchell PB Niven H Finch A Wedgwood L Scimone A Blair IP Parker G Schofield PR Life events, first depression onset and the serotonin transporter gene Br J Psychiatry 2006 188 210 215 10.1192/bjp.bp.105.009522 16507960

[B19] Cervilla JA Molina E Rivera M Torres-González F Bellón JA Moreno B Luna JD Lorente JA Mayoral F King M Nazaret I Gutiérrez B PREDICT Study Core Group The risk for depression conferred by stressful life events is modified by variation at the serotonin transporter 5HTTLPR genotype: evidence from the Spanish PREDICT-Gene cohort Mol Psychiatry 2007 12 8 748 755 10.1038/sj.mp.4001981 17387319

[B20] Kaufman J Yang BZ Douglas-Palumberi H Houshyar S Lipschitz D Krystal JH Gelernter J Social supports and serotonin transporter gene moderate depression in maltreated children Proc Natl Acad Sci USA 2004 101 49 17316 17321 10.1073/pnas.0404376101 15563601 PMC534414

[B21] Kendler KS Kuhn JW Vittum J Prescott CA Riley B The interaction of stressful life events and a serotonin transporter polymorphism in the prediction of episodes of major depression: a replication Arch Gen Psychiatry 2005 62 5 529 535 10.1001/archpsyc.62.5.529 15867106

[B22] Eley TC Sugden K Corsico A Gregory AM Sham P McGuffin P Plomin R Craig IW Gene-environment interaction analysis of serotonin system markers with adolescent depression Mol Psychiatry 2004 9 10 908 915 10.1038/sj.mp.4001546 15241435

[B23] Grabe HJ Lange M Wolff B Völzke H Lucht M Freyberger HJ John U Cascorbi I Mental and physical distress is modulated by a polymorphism in the 5-HT transporter gene interacting with social stressors and chronic disease burden Mol Psychiatry 2005 10 2 220 224 10.1038/sj.mp.4001555 15263905

[B24] Taylor SE Way BM Welch WT Hilmert CJ Lehman BJ Eisenberger NI Early family environment, current adversity, the serotonin transporter promoter polymorphism, and depressive symptomatology Biol Psychiatry 2006 60 7 671 676 10.1016/j.biopsych.2006.04.019 16934775

[B25] Gillespie NA Whitfield JB Williams B Heath AC Martin NG The relationship between stressful life events, the serotonin transporter (5-HTTLPR) genotype and major depression Psychol Med 2005 35 1 101 111 10.1017/S0033291704002727 15842033

[B26] Surtees PG Wainwright NW Willis-Owen SA Luben R Day NE Flint J Social adversity, the serotonin transporter (5-HTTLPR) polymorphism and major depressive disorder Biol Psychiatry 2006 59 3 224 229 10.1016/j.biopsych.2005.07.014 16154545

[B27] Chipman P Jorm AF Prior M Sanson A Smart D Tan X Easteal S No interaction between the serotonin transporter polymorphism (5-HTTLPR) and childhood adversity or recent stressful life events on symptoms of depression: results from two community surveys Am J Med Genet B Neuropsychiatr Genet 2007 144B 4 561 565 10.1002/ajmg.b.30480 17450557

[B28] Chorbov VM Lobos EA Todorov AA Heath AC Botteron KN Todd RD Relationship of 5-HTTLPR genotypes and depression risk in the presence of trauma in a female twin sample Am J Med Genet B Neuropsychiatr Genet 2007 144B 6 830 833 10.1002/ajmg.b.30534 17455215

[B29] Hankin BL Jenness J Abela JR Smolen A Interaction of 5-HTTLPR and idiographic stressors predicts prospective depressive symptoms specifically among youth in a multiwave design J Clin Child Adolesc Psychol 2011 40 4 572 585 10.1080/15374416.2011.581613 21722029 PMC3164979

[B30] Nobile M Rusconi M Bellina M Marino C Giorda R Carlet O Vanzin L Molteni M Battaglia M The influence of family structure, the TPH2 G-703 T and the 5-HTTLPR serotonergic genes upon affective problems in children aged 10–14 years J Child Psychol Psychiatry 2009 50 3 317 325 10.1111/j.1469-7610.2008.01958.x 19175813

[B31] Hoefgen B Schulze TG Ohlraun S von Widdern O Höfels S Gross M Heidmann V Kovalenko S Eckermann A Kölsch H Metten M Zobel A Becker T Nöthen MM The power of sample size and homogenous sampling: association between the 5-HTTLPR serotonin transporter polymorphism and major depressive disorder Biol Psychiatry 2005 57 3 247 251 10.1016/j.biopsych.2004.11.027 15691525

[B32] Clarke H Flint J Attwood AS Munafò MR Association of the 5- HTTLPR genotype and unipolar depression: a meta-analysis Psychol Med 2010 40 11 1767 1778 10.1017/S0033291710000516 20380781

[B33] Cervilla JA Rivera M Molina E Torres-González F Bellón JA Moreno B de Dios LJ Lorente JA de Diego-Otero Y King M Nazareth I Gutiérrez B PREDICT Study Core Group The 5-HTTLPR s/s genotype at the serotonin transporter gene (SLC6A4) increases the risk for depression in a large cohort of primary care attendees: the PREDICT-gene study Am J Med Genet B Neuropsychiatr Genet 2006 141B 8 912 917 10.1002/ajmg.b.30455 17063469

[B34] Kiyohara C Yoshimasu K Association between major depressive disorder and a functional polymorphism of the 5-hydroxytryptamine (serotonin) transporter gene: a meta-analysis Psychiatr Genet 2010 20 2 49 58 10.1097/YPG.0b013e328335112b 20016401

[B35] Martinowich K Lu B Interaction between BDNF and serotonin: role in mood disorders Neuropsychopharmacology 2008 33 1 73 83 10.1038/sj.npp.1301571 17882234

[B36] Egan MF Kojima M Callicott JH Goldberg TE Kolachana BS Bertolino A Zaitsev E Gold B Goldman D Dean M Lu B Weinberger DR The BDNF val66met polymorphism affects activity-dependent secretion of BDNF and human memory and hippocampal function Cell 2003 112 2 257 269 10.1016/S0092-8674(03)00035-7 12553913

[B37] Hwang JP Tsai SJ Hong CJ Yang CH Lirng JF Yang YM The Val66Met polymorphism of the brain-derived neurotrophic-factor gene is associated with geriatric depression Neurobiol Aging 2006 27 12 1834 1837 10.1016/j.neurobiolaging.2005.10.013 16343697

[B38] Strauss J Barr CL George CJ Devlin B Vetró A Kiss E Baji I King N Shaikh S Lanktree M Kovacs M Kennedy JL Brain-derived neurotrophic factor variants are associated with childhood-onset mood disorder: confirmation in a Hungarian sample Mol Psychiatry 2005 10 9 861 867 10.1038/sj.mp.4001685 15940299

[B39] Surtees PG Wainwright NW Willis-Owen SA Sandhu MS Luben R Day NE Flint J No association between the BDNF Val66Met polymorphism and mood status in a non-clinical community sample of 7389 older adults J Psychiatr Res 2007 41 5 404 409 10.1016/j.jpsychires.2006.01.004 16497333

[B40] Kaufman J Yang BZ Douglas-Palumberi H Grasso D Lipschitz D Houshyar S Krystal JH Gelernter J Brain-derived neurotrophic factor-5-HTTLPR gene interactions and environmental modifiers of depression in children Biol Psychiatry 2006 59 8 673 680 10.1016/j.biopsych.2005.10.026 16458264

[B41] Aguilera M Arias B Wichers M Barrantes-Vidal N Moya J Villa H van Os J Ibanez MI Ruiperez MA Ortet G Fananas L Early adversity and 5-HTT/BDNF genes: new evidence of gene-environment interactions on depressive symptoms in a general population Psychol Med 2009 39 9 1425 1432 10.1017/S0033291709005248 19215635

[B42] Grabe HJ Schwahn C Mahler J Appel K Schulz A Spitzer C Fenske K Barnow S Freyberger HJ Teumer A Petersmann A Biffar R Rosskopf D John U Volzke H Genetic epistasis between the brain-derived neurotrophic factor Val66Met polymorphism and the 5-HTT promoter polymorphism moderates the susceptibility to depressive disorders after childhood abuse Prog Neuropsychopharmacol Biol Psychiatry 2012 36 2 264 270 10.1016/j.pnpbp.2011.09.010 21996278

[B43] Nederhof E Bouma EM Oldehinkel AJ Ormel J Interaction between childhood adversity, brain-derived neurotrophic factor val/met and serotonin transporter promoter polymorphism on depression: the TRAILS study Biol Psychiatry 2010 68 2 209 212 10.1016/j.biopsych.2010.04.006 20553751

[B44] Wichers M Kenis G Jacobs N Mengelers R Derom C Vlietinck R van Os J The BDNF Val(66)Met x 5-HTTLPR x child adversity interaction and depressive symptoms: An attempt at replication Am J Med Genet B Neuropsychiatr Genet 2008 147B 1 120 123 10.1002/ajmg.b.30576 17579366

[B45] Cederblad M Höök B Berg R [Screening of psychosocial risk factors during infancy and childhood] Socialmedicinsk tidskrift 2005 82 158 170 In Swedish

[B46] Cederblad M Höök B [Psychosocial health among second-generation immigrant children in preschool age: risk- and resilient factors] Psykosocial hälsa hos andra generationens invandrarbarn under förskoleåren: risk- och friskfaktorer Socialmedicinsk tidskrift 2006 83 217 229 In Swedish

[B47] Höök B Cederblad M Berg R Prenatal and postnatal maternal smoking as risk factors for preschool children’s mental health Acta Paediatr 2006 95 671 677 10.1080/08035250500538965 16754547

[B48] Dekeyser L Svedin CG Agnafors S Sydsjö G Self-reported mental health in 12-year-old second-generation immigrant children in Sweden Nord J Psychiatry 2011 65 6 389 395 10.3109/08039488.2011.566936 21417579

[B49] Comasco E Sylvén SM Papadopoulos FC Oreland L Sundström-Poromaa I Skalkidou A Postpartum depressive symptoms and the BDNF Val66Met functional polymorphism: effect of season of delivery Arch Womens Ment Health 2011 14 6 453 463 10.1007/s00737-011-0239-x 21997575

[B50] Cox JL Holden JM Sagovsky R Detection of postnatal depression. Development of the 10-item Edinburgh Postnatal Depression Scale Br J Psychiatry 1987 150 782 786 10.1192/bjp.150.6.782 3651732

[B51] Nordberg L Rydelius PA Nylander I Aurelius G Zetterström R Psychomotor and mental development during infancy. Relation to psychosocial conditions and health. Part IV of a longitudinal study of children in a new Stockholm suburb Acta Paediatr Scand Suppl 1989 353 1 35 2801111 10.1111/j.1651-2227.1989.tb11228.x

[B52] Achenbach TM Manual for the Child Behavior Checklist/4–18 and 1991 Profile1991a Burlington, VT: Department of Psychiatry, University of Vermont

[B53] Derogatis LR Lipmann RS Rickels K The Hopkins symptoms checklist (HSCL): a self-report inventory Behav Sci 1974 19 1 15 10.1002/bs.3830190102 4808738

[B54] Greenwald R Rubin A Brief assessment of children’s post-traumatic symptoms: Development and preliminary validation of parent and child scales Res Soc Work Practice 1999 9 61 75 10.1177/104973159900900105

[B55] Larsson I LITE-P, life incidence of traumatic events. (Translation into Swedish, with permission from the author Greenwald R.) 2003 Linköping: Linköping University

[B56] Matthey S Henshaw C Elliot S Barnett B Variability in use of cut-off scores and formats on the Edinburgh Postnatal Depression Scale – implications for clinical and research practice Arch Womens Ment Health 2006 9 309 315 10.1007/s00737-006-0152-x 17013761

[B57] Nettelbladt P Hansson L Stefansson CG Borgquist L Nordström G Test characteristics of the Hopkins symptom check list-25 (HSCL-25) in Sweden, using the present state examination (PSE-9) as a caseness criterion Soc Psychiatry Psychiatr Epidemiol 1993 28 3 130 133 10.1007/BF00801743 8378808

[B58] Duncan LE Keller MC A critical review of the first 10 years of candidate gene-by-environment interaction research in psychiatry Am J Psychiatry 2011 168 10 1041 1049 10.1176/appi.ajp.2011.11020191 21890791 PMC3222234

[B59] Thomson CC Roberts K Curran A Ryan L Wright RJ Caretaker-child concordance for child’s exposure to violence in a pre-adolescent inner-city population Arch Pediatr Adolesc Med 2002 156 818 823 12144374 10.1001/archpedi.156.8.818

[B60] Angold A Costello EJ Erkanli A Comorbidity J Child Psychol Psychiatry 1999 40 1 57 87 10.1111/1469-7610.00424 10102726

[B61] Laucht M Treutlein J Blomeyer D Buchmann AF Schmid B Becker K Zimmermann US Schmidt MH Esser G Rietschel M Banaschewski T Interaction between the 5-HTTLPR serotonin transporter polymorphism and environmental adversity for mood and anxiety psychopathology: evidence from a high-risk community sample of young adults Int J Neuropsychopharmacol 2009 12 6 737 747 10.1017/S1461145708009875 19154632

[B62] van der Toorn SL Huizink AC Utens EM Verhulst FC Ormel J Ferdinand RF Maternal depressive symptoms, and not anxiety symptoms, are associated with positive mother-child reporting discrepancies of internalizing problems in children: a report on the TRAILS study Eur Child Adolesc Psychiatry 2010 19 4 379 388 10.1007/s00787-009-0062-3 19823897 PMC2843837

[B63] Feder A Nestler EJ Charney DS Psychobiology and molecular genetics of resilience Nat Rev Neurosci 2009 10 6 446 457 10.1038/nrn2649 19455174 PMC2833107

