# Supplementary material for: Effect of gene, environment and maternal depressive symptoms on pre-adolescence behavior problems – a longitudinal study
Source: Child Adolesc Psychiatry Ment Health. 2013 Mar 22;7:10. doi: 10.1186/1753-2000-7-10 (PMC3615948; doi:10.1186/1753-2000-7-10)
Supplement: Additional file 1 — Factors and interactions included in multivariate analysis. The additional file holds a list of all factors and interactions between factors tested in multivariate analysis. [file 1753-2000-7-10-S1.doc]

**Factors and interactions included in multivariate analysis**

EPDS

HSCL-25

*BDNF* Val66Met

*5-*HTTLPR

LITE

Ethnical background

Sex

LSS

Interaction *BDNF* + EPDS

Interaction *BDNF* + HSCL

Interaction *BDNF* + LITE

Interaction *BDNF* + Ethnical background

Interaction *BDNF* + *5-*HTTLPR

Interaction HSCL-25 + EPDS

Interaction *5-*HTTLPR + EPDS

Interaction *5-*HTTLPR + HSCL-25

Interaction *5-*HTTLPR + LITE

Interaction *5-*HTTLPR + Ethnical background

Interaction *BDNF* + *5-*HTTLPR + LITE

Note: EPDS = Edinburgh Postnatal Depression Scale, HSCL-25 = Hopkins Symptom Checklist 25, *BDNF =* Brain Derived Neurotrophic Factor, 5-HTTLPR = serotonin transporter gene-linked polymorphic region, LITE = Life Incidence of Traumatic Events, LSS = Life Stress Score
